# (2-*tert*-Butyl-3-phenyl-2,3-di­hydro­isoxazole-4,5-di­yl)bis­(phenyl­methanone)

**DOI:** 10.1107/S1600536813019508

**Published:** 2013-07-20

**Authors:** R. Sandhya, M. Sithambaresan, S. Prathapan, M. R. Prathapachandra Kurup

**Affiliations:** a Department of Applied Chemistry, Cochin University of Science and Technology, Kochi 682 022, India; b Department of Chemistry, Faculty of Science, Eastern University, Sri Lanka, Chenkalady, Sri Lanka

## Abstract

The phenyl and *tert*-butyl groups of the title compound, C_27_H_25_NO_3_, exhibit a *trans* configuration in agreement with the stereochemistry of the *Z* phenyl-*N*-*tert*-butyl­nitrone starting material. The attached carbonyl groups are not coplanar with the neighboring di­hydro­isoxazole ring and the phenyl rings they are bonded to, with torsion angles of 59.26 (8), 17.53 (11), 16.52 (12) and 52.86 (7)°. The dihedral angle between the di­hydro­isoxazole ring and the directly attached phenyl group is 86.86 (8)°. There are two nonclassical inter­molecular C—H⋯O hydrogen-bonding inter­actions that operate together with an inter­molecular C—H⋯π inter­action to form a supramolecular architecture in the crystal system.

## Related literature

For background to isoxazoline derivatives and their applications, see: Kiss *et al.* (2009 ▶); Velikorodov & Sukhenko (2003 ▶); Shi *et al.* (2012 ▶); Khan & Lee (2006 ▶). For the mechanism of the 1,3-dipolar cyclo­addition of nitro­nes with alkynes, see: Eberson *et al.* (1998 ▶). For the synthesis of related compounds, see: Chakraborty *et al.* (2012 ▶).
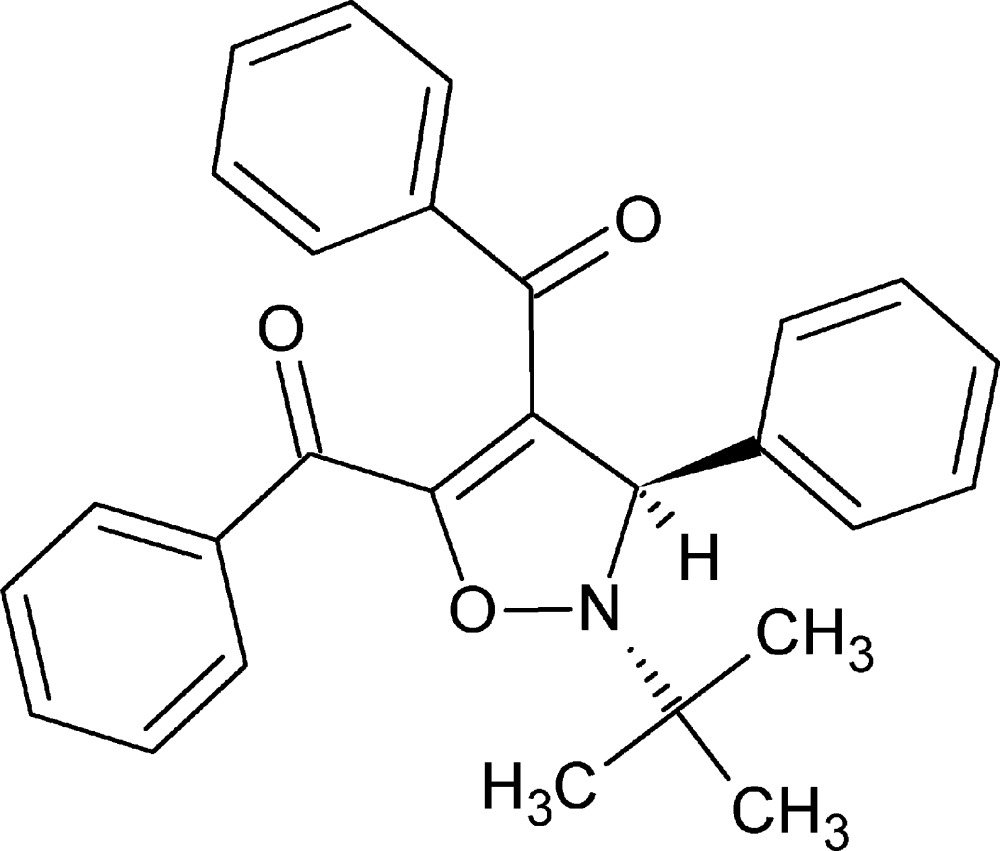


## Experimental

### 

#### Crystal data

C_27_H_25_NO_3_
*M* *_r_* = 411.48
Orthorhombic, 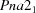

*a* = 20.1034 (12) Å
*b* = 17.799 (1) Å
*c* = 6.1366 (3) Å
*V* = 2195.8 (2) Å^3^
*Z* = 4
Mo *K*α radiation
μ = 0.08 mm^−1^
*T* = 296 K
0.40 × 0.20 × 0.20 mm

#### Data collection

Bruker Kappa APEXII CCD diffractometer
Absorption correction: multi-scan (*SADABS*; Bruker, 2004 ▶) *T*_min_ = 0.968, *T*_max_ = 0.984
20181 measured reflections
2967 independent reflections
2422 reflections with *I* > 2σ(*I*)
*R*_int_ = 0.025

#### Refinement

*R*[*F* ^2^ > 2σ(*F* ^2^)] = 0.038
*wR*(*F* ^2^) = 0.094
*S* = 1.05
2967 reflections
283 parameters
1 restraint
H-atom parameters constrained
Δρ_max_ = 0.11 e Å^−3^
Δρ_min_ = −0.16 e Å^−3^

### 

Data collection: *APEX2* (Bruker, 2004 ▶); cell refinement: *APEX2* and *SAINT* (Bruker, 2004 ▶); data reduction: *SAINT* and *XPREP* (Bruker, 2004 ▶); program(s) used to solve structure: *SHELXS97* (Sheldrick, 2008 ▶); program(s) used to refine structure: *SHELXL97* (Sheldrick, 2008 ▶); molecular graphics: *ORTEP-3 for Windows* (Farrugia, 2012 ▶) and *DIAMOND* (Brandenburg, 2010 ▶); software used to prepare material for publication: *SHELXL97* and *publCIF* (Westrip, 2010 ▶).

## Supplementary Material

Crystal structure: contains datablock(s) I, global. DOI: 10.1107/S1600536813019508/zl2560sup1.cif

Structure factors: contains datablock(s) I. DOI: 10.1107/S1600536813019508/zl2560Isup2.hkl

Supplementary material file. DOI: 10.1107/S1600536813019508/zl2560Isup3.cml

Additional supplementary materials:  crystallographic information; 3D view; checkCIF report

## Figures and Tables

**Table 1:** Hydrogen-bond geometry (Å, °) *Cg*1 is the centroid of the C18–C23 ring.

*D*—H⋯*A*	*D*—H	H⋯*A*	*D*⋯*A*	*D*—H⋯*A*
C3—H3⋯O3^i^	0.93	2.51	3.203 (3)	131
C25—H25*A*⋯O1^ii^	0.96	2.59	3.483 (3)	155
C2—H2⋯*Cg*1^i^	0.93	2.70	3.490 (3)	143

Symmetry codes: (i) 
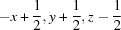
; (ii) 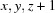.

**Fig. 1.**
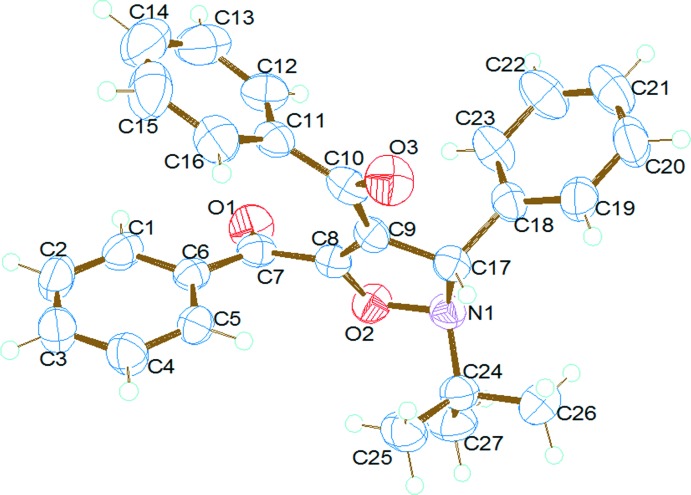
ORTEP view of the title compound drawn with 50% probability displacement ellipsoids for the non-H atoms.

**Fig. 2.**
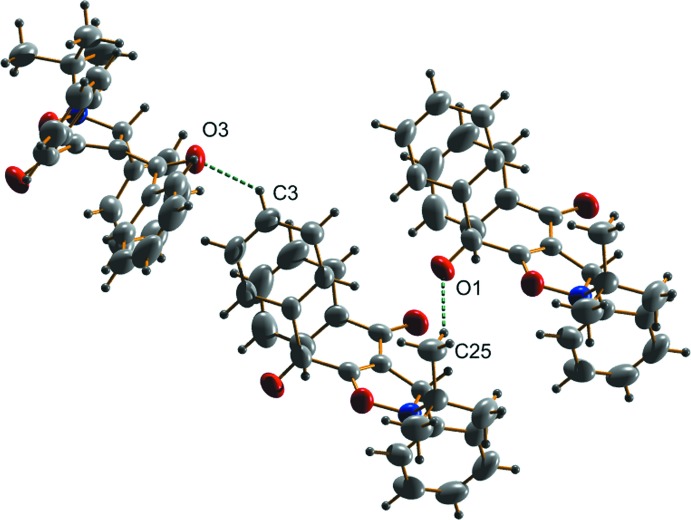
C—H···O intermolecular hydrogen bonding interactions found in the title compound.

**Fig. 3.**
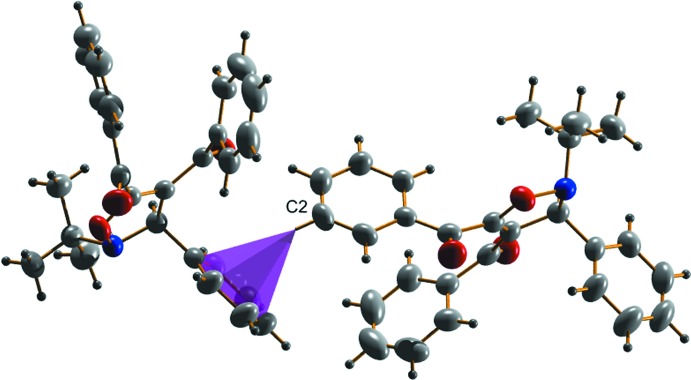
C—H···π interaction found in the compound C27H25NO3.

**Fig. 4.**
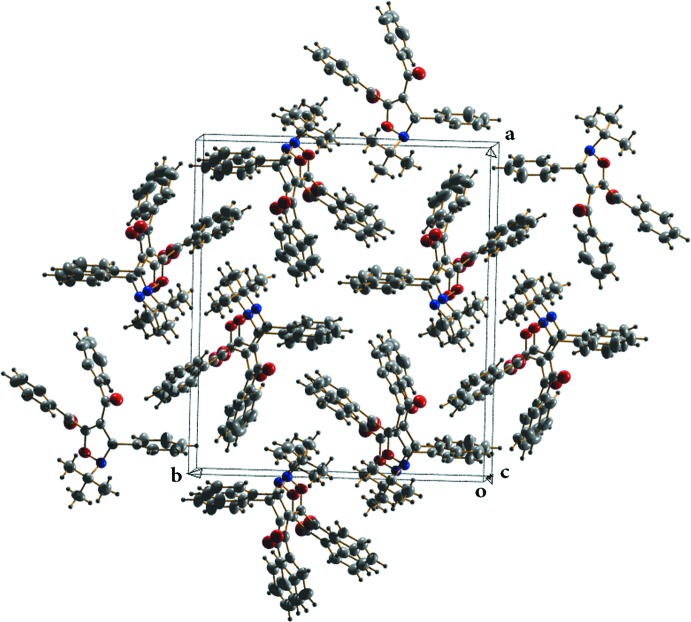
Packing diagram of the compound along the c axis.

## supplementary crystallographic information

### Comment

Isoxazolines are an important class of heterocyclic compounds because of their wide variety of applications. In chemistry, they find use as intermediates in organic synthesis (Kiss *et al.*, 2009), and many isoxazoline derivatives are bilologocally active compounds with antimicrobial, anticancer, analgesic and anti-inflammatory properties (Velikorodov and Sukhenko, 2003; Shi *et al.*, 2012; Khan and Lee, 2006). Considering these applications we report the structure of a 4-isoxazoline derivative, which was prepared by the 1,3-dipolar cycloaddition reaction of phenyl-N-*tert*-butylnitrone with dibenzoylacetylene.

The compound (Fig. 1) crystallizes in the orthorhombic space group *P*na2_1_. The torsion angle of 125.52 (18)° of the molecular fragment C24/N1/C17/C18 shows the phenyl and tert butyl groups be *trans* to each other, which agrees with the stereochemistry of the Z phenyl-N-*tert*-butylnitrone starting material based on the mechanism of the reaction (Eberson *et al.*, 1998). The dihedral angle of the dihydroisoxazole ring with the directly attached phenyl group is 86.86 (8)°. The carbonyl groups attached are not coplanar with the neighboring dihydroisoxazole ring and the phenyl rings they are bonded to. The carbonyl group between the C1–C6 phenyl ring and the dihydroisoxazole ring significantly deviates from the average planes of the phenyl and the dihydroisoxazole rings, respectively, with the largest deviations being 0.2065 (1) and 0.4366 (1) Å for the O1 atom. The torsion angles between the plane of the carbonyl group (atoms C6, C7, O1 and C8) and those of the phenyl and the dihydroisoxazole rings are 17.53 (11) and 59.26 (8)° respectively. The other carbonyl group between the C11-C16 phenyl ring and the dihydroisoxazole ring also significantly deviates from the planes of the attached phenyl and the dihydroisoxazole rings, respectively, with the largest deviations being 0.4777 (1) and 0.1441 (1) Å for the O3 atom. The torsion angles between the plane of the carbonyl group (atoms C9, C10, O3 and C11) and those of the phenyl and the dihydroisoxazole rings are 52.86 (7) and 16.52 (12)° respectively.

There are two intermolecular C–H···O hydrogen bond interactions (Fig. 2) between the H atoms attached at the C3 & C25 and O3 & O1 atoms of neighboring molecules with D···A distances of 3.203 (3) and 3.483 (3) Å, respectively. An intermolecular C–H···π interaction (Fig. 3) between the H at C2 and the C18-C23 aromatic ring of an adjacent molecule with an H···π distance of 2.70 Å also supports the interconnection between the molecules. Thus, these intermolecular hydrogen bonding interactions, augmented by a weak C–H···π interaction, play a major role in the formation of the supramolecular network of the molecular units. Fig. 4 shows a packing diagram of the title compound viewed along the c axis direction.

### Experimental

The title compound was prepared by adapting a reported procedure (Chakraborty *et al.*, 2012). Phenyl-N-*tert*-butylnitrone (3 mmol) and dibenzoylacetylene (3 mmol) were added into 15 mL of acetonitrile and stirred for 4 h at room temperature. The reaction was monitored by TLC using EtOAc/hexane (1:5). The solvent was removed under reduced pressure and the product was purified from the crude by column chromatography on silica gel. Yellow crystals suitable for X-ray structure determination were grown from ethanol by slow evaporation (m.p: 110 °C).

IR (KBr, υ in cm^-1^): 3054, 2970, 1655, 1624, 1439, 1355, 1294, 1186, 1063,955,855,770, 694, 525. ^1^H NMR (400 MHz, CDCl_3_, δ, ppm): 1.27 (s, 9H), 6.0 (s, 1H), 7.05-7.74 (m, 15H). ^13^C NMR (400 MHz, CDCl_3_, δ, p.p.m.): 190.61, 185.68, 155.93, 142.29, 139.60, 135.52, 134.35, 132.00, 129.23, 128.71, 128.63, 128.01, 127.72, 127.49, 120.31, 69.53, 61.83, 24.99. MS: *m/z* calculated for C_27_H_25_NO_3_: 411 (M^+^); *m/z* measured: 411(M^+^), 412 (M+1).

### Refinement

All H atoms on C were placed in calculated positions, guided by difference maps, with C–H bond distances of 0.93–0.98 Å. H atoms were assigned *U*_iso_ = 1.2U_eq_(carrier) or 1.5U_eq_ (methyl C). Omitted owing to bad disagreement were the reflections (1 0 0), (2 0 0) and (1 1 0). In the absence of significant anomalous scattering effects, Friedel pairs have been merged.

### Crystal data

**Table:**

C_27_H_25_NO_3_	*F*(000) = 872
*M**_r_* = 411.48	*D*_x_ = 1.245 Mg m^−^^3^
Orthorhombic, *P**n**a*2_1_	Mo *K*α radiation, λ = 0.71073 Å
Hall symbol: P 2c -2n	Cell parameters from 8059 reflections
*a* = 20.1034 (12) Å	θ = 2.3–25.1°
*b* = 17.799 (1) Å	µ = 0.08 mm^−^^1^
*c* = 6.1366 (3) Å	*T* = 296 K
*V* = 2195.8 (2) Å^3^	Block, yellow
*Z* = 4	0.40 × 0.20 × 0.20 mm

### Data collection

**Table:**

Bruker Kappa APEXII CCD diffractometer	2967 independent reflections
Radiation source: fine-focus sealed tube	2422 reflections with *I* > 2σ(*I*)
Graphite monochromator	*R*_int_ = 0.025
Detector resolution: 8.33 pixels mm^-1^	θ_max_ = 28.3°, θ_min_ = 3.1°
ω and φ scan	*h* = −26→24
Absorption correction: multi-scan (*SADABS*; Bruker, 2004)	*k* = −23→20
*T*_min_ = 0.968, *T*_max_ = 0.984	*l* = −8→8
20181 measured reflections	

### Refinement

**Table:**

Refinement on *F*^2^	Primary atom site location: structure-invariant direct methods
Least-squares matrix: full	Secondary atom site location: difference Fourier map
*R*[*F*^2^ > 2σ(*F*^2^)] = 0.038	Hydrogen site location: inferred from neighbouring sites
*wR*(*F*^2^) = 0.094	H-atom parameters constrained
*S* = 1.05	*w* = 1/[σ^2^(*F*_o_^2^) + (0.0417*P*)^2^ + 0.2966*P*] where *P* = (*F*_o_^2^ + 2*F*_c_^2^)/3
2967 reflections	(Δ/σ)_max_ < 0.001
283 parameters	Δρ_max_ = 0.11 e Å^−^^3^
1 restraint	Δρ_min_ = −0.16 e Å^−^^3^

### Special details

**Table:**

Geometry. All esds (except the esd in the dihedral angle between two l.s. planes) are estimated using the full covariance matrix. The cell esds are taken into account individually in the estimation of esds in distances, angles and torsion angles; correlations between esds in cell parameters are only used when they are defined by crystal symmetry. An approximate (isotropic) treatment of cell esds is used for estimating esds involving l.s. planes.
Refinement. Refinement of F^2^ against ALL reflections. The weighted R-factor wR and goodness of fit S are based on F^2^, conventional R-factors R are based on F, with F set to zero for negative F^2^. The threshold expression of F^2^ > 2sigma(F^2^) is used only for calculating R-factors(gt) etc. and is not relevant to the choice of reflections for refinement. R-factors based on F^2^ are statistically about twice as large as those based on F, and R- factors based on ALL data will be even larger.

### Fractional atomic coordinates and isotropic or equivalent isotropic displacement parameters (Å^2^)

**Table:**

	*x*	*y*	*z*	*U*_iso_*/*U*_eq_	
O1	0.14603 (9)	0.40278 (10)	0.0012 (3)	0.0661 (5)	
O2	0.05003 (7)	0.36111 (8)	0.3046 (3)	0.0517 (4)	
O3	0.20313 (8)	0.27030 (9)	0.7795 (3)	0.0616 (4)	
N1	0.01783 (8)	0.30875 (9)	0.4612 (3)	0.0458 (4)	
C3	0.25597 (12)	0.60359 (13)	0.4233 (5)	0.0625 (7)	
H3	0.2772	0.6473	0.4690	0.075*	
C2	0.26649 (13)	0.57720 (14)	0.2177 (5)	0.0637 (7)	
H2	0.2956	0.6023	0.1250	0.076*	
C1	0.23438 (11)	0.51380 (13)	0.1469 (4)	0.0545 (5)	
H1	0.2416	0.4962	0.0062	0.065*	
C6	0.19103 (9)	0.47575 (10)	0.2851 (3)	0.0402 (4)	
C7	0.15240 (10)	0.41275 (12)	0.1949 (4)	0.0446 (5)	
C8	0.11570 (9)	0.36106 (10)	0.3468 (3)	0.0407 (4)	
C9	0.13418 (9)	0.31458 (10)	0.5061 (3)	0.0398 (4)	
C17	0.07315 (9)	0.27504 (10)	0.5896 (4)	0.0432 (4)	
H17	0.0670	0.2856	0.7451	0.052*	
C18	0.07424 (9)	0.19093 (11)	0.5505 (4)	0.0456 (5)	
C23	0.09136 (13)	0.16269 (13)	0.3489 (4)	0.0619 (6)	
H23	0.1022	0.1956	0.2367	0.074*	
C22	0.09257 (16)	0.08604 (14)	0.3114 (5)	0.0763 (8)	
H22	0.1048	0.0677	0.1752	0.092*	
C21	0.07586 (14)	0.03747 (13)	0.4739 (6)	0.0761 (8)	
H21	0.0765	−0.0140	0.4486	0.091*	
C24	−0.03126 (10)	0.35530 (11)	0.5852 (4)	0.0510 (5)	
C26	−0.06460 (13)	0.30225 (15)	0.7463 (5)	0.0782 (9)	
H26A	−0.0784	0.2575	0.6717	0.117*	
H26B	−0.1027	0.3265	0.8091	0.117*	
H26C	−0.0337	0.2893	0.8595	0.117*	
C27	−0.08187 (12)	0.38226 (15)	0.4187 (5)	0.0708 (7)	
H27A	−0.0603	0.4140	0.3139	0.106*	
H27B	−0.1163	0.4100	0.4912	0.106*	
H27C	−0.1010	0.3397	0.3457	0.106*	
C25	−0.00051 (13)	0.42144 (13)	0.7032 (5)	0.0639 (6)	
H25A	0.0318	0.4036	0.8061	0.096*	
H25B	−0.0346	0.4488	0.7788	0.096*	
H25C	0.0208	0.4539	0.5996	0.096*	
C20	0.05818 (14)	0.06446 (14)	0.6739 (6)	0.0737 (8)	
H20	0.0466	0.0312	0.7845	0.088*	
C19	0.05747 (11)	0.14130 (13)	0.7128 (5)	0.0595 (6)	
H19	0.0456	0.1593	0.8497	0.071*	
C10	0.19933 (10)	0.30137 (10)	0.6033 (4)	0.0438 (4)	
C11	0.26076 (9)	0.32369 (10)	0.4850 (4)	0.0451 (5)	
C16	0.30928 (11)	0.36438 (13)	0.5935 (5)	0.0619 (6)	
H16	0.3019	0.3810	0.7351	0.074*	
C15	0.36829 (14)	0.37991 (17)	0.4906 (7)	0.0864 (10)	
H15	0.4003	0.4087	0.5612	0.104*	
C14	0.38057 (15)	0.35349 (19)	0.2856 (8)	0.0933 (11)	
H14	0.4213	0.3629	0.2193	0.112*	
C13	0.33269 (16)	0.31311 (17)	0.1776 (5)	0.0814 (9)	
H13	0.3410	0.2951	0.0380	0.098*	
C12	0.27250 (12)	0.29918 (13)	0.2752 (4)	0.0576 (6)	
H12	0.2396	0.2732	0.1998	0.069*	
C5	0.18161 (10)	0.50193 (11)	0.4950 (4)	0.0462 (4)	
H5	0.1534	0.4764	0.5897	0.055*	
C4	0.21427 (12)	0.56621 (12)	0.5637 (4)	0.0560 (5)	
H4	0.2080	0.5840	0.7047	0.067*	

### Atomic displacement parameters (Å^2^)

**Table:**

	*U*^11^	*U*^22^	*U*^33^	*U*^12^	*U*^13^	*U*^23^
O1	0.0854 (12)	0.0756 (10)	0.0372 (8)	−0.0054 (9)	−0.0025 (8)	−0.0026 (8)
O2	0.0428 (7)	0.0611 (8)	0.0511 (8)	0.0062 (6)	−0.0059 (7)	0.0124 (7)
O3	0.0648 (9)	0.0699 (10)	0.0502 (9)	0.0031 (7)	−0.0153 (8)	0.0129 (8)
N1	0.0422 (8)	0.0427 (8)	0.0526 (10)	0.0021 (7)	−0.0033 (8)	0.0006 (8)
C3	0.0596 (14)	0.0528 (12)	0.0750 (18)	−0.0103 (10)	0.0015 (13)	0.0032 (13)
C2	0.0545 (13)	0.0655 (14)	0.0711 (17)	−0.0108 (11)	0.0147 (12)	0.0140 (13)
C1	0.0516 (12)	0.0644 (13)	0.0475 (12)	0.0049 (10)	0.0121 (10)	0.0053 (10)
C6	0.0397 (9)	0.0416 (9)	0.0393 (10)	0.0089 (7)	0.0017 (9)	0.0072 (8)
C7	0.0485 (11)	0.0460 (10)	0.0394 (11)	0.0100 (8)	−0.0002 (9)	0.0031 (9)
C8	0.0419 (10)	0.0406 (10)	0.0398 (11)	0.0025 (8)	−0.0046 (8)	−0.0043 (8)
C9	0.0437 (9)	0.0364 (8)	0.0393 (10)	−0.0005 (7)	−0.0060 (9)	−0.0032 (8)
C17	0.0467 (10)	0.0427 (10)	0.0402 (10)	0.0024 (8)	−0.0020 (9)	0.0009 (9)
C18	0.0425 (10)	0.0397 (9)	0.0544 (13)	−0.0026 (8)	−0.0094 (9)	0.0057 (9)
C23	0.0876 (17)	0.0436 (11)	0.0546 (15)	−0.0034 (11)	−0.0131 (13)	0.0018 (11)
C22	0.109 (2)	0.0506 (13)	0.0698 (17)	−0.0003 (14)	−0.0221 (17)	−0.0089 (13)
C21	0.0867 (18)	0.0427 (12)	0.099 (2)	−0.0096 (12)	−0.0261 (18)	0.0003 (15)
C24	0.0430 (10)	0.0489 (11)	0.0611 (13)	0.0021 (9)	0.0018 (10)	−0.0082 (11)
C26	0.0658 (15)	0.0703 (15)	0.098 (2)	−0.0048 (12)	0.0304 (16)	−0.0025 (16)
C27	0.0476 (12)	0.0747 (15)	0.090 (2)	0.0146 (11)	−0.0092 (13)	−0.0145 (16)
C25	0.0631 (14)	0.0559 (13)	0.0727 (17)	0.0017 (11)	−0.0043 (13)	−0.0172 (13)
C20	0.0683 (15)	0.0519 (14)	0.101 (2)	−0.0095 (12)	−0.0033 (16)	0.0288 (15)
C19	0.0527 (12)	0.0575 (13)	0.0684 (16)	−0.0018 (10)	0.0030 (11)	0.0151 (12)
C10	0.0524 (11)	0.0374 (9)	0.0415 (10)	0.0045 (8)	−0.0131 (9)	−0.0050 (9)
C11	0.0434 (10)	0.0399 (10)	0.0520 (12)	0.0079 (8)	−0.0102 (10)	0.0036 (9)
C16	0.0570 (13)	0.0583 (13)	0.0704 (16)	−0.0015 (10)	−0.0167 (13)	0.0024 (13)
C15	0.0545 (15)	0.0826 (19)	0.122 (3)	−0.0141 (13)	−0.0181 (19)	0.025 (2)
C14	0.0583 (16)	0.098 (2)	0.123 (3)	0.0151 (16)	0.018 (2)	0.045 (2)
C13	0.0803 (19)	0.0897 (19)	0.0742 (19)	0.0328 (16)	0.0175 (16)	0.0174 (16)
C12	0.0582 (13)	0.0591 (13)	0.0556 (14)	0.0142 (10)	−0.0043 (11)	−0.0019 (11)
C5	0.0490 (11)	0.0467 (10)	0.0428 (10)	−0.0020 (8)	0.0056 (10)	0.0041 (10)
C4	0.0655 (13)	0.0510 (11)	0.0513 (13)	−0.0036 (10)	0.0040 (11)	−0.0052 (10)

### Geometric parameters (Å, º)

**Table:**

O1—C7	1.209 (3)	C24—C27	1.520 (4)
O2—C8	1.345 (2)	C24—C26	1.522 (3)
O2—N1	1.487 (2)	C26—H26A	0.9600
O3—C10	1.217 (3)	C26—H26B	0.9600
N1—C17	1.489 (2)	C26—H26C	0.9600
N1—C24	1.496 (3)	C27—H27A	0.9600
C3—C2	1.363 (4)	C27—H27B	0.9600
C3—C4	1.374 (3)	C27—H27C	0.9600
C3—H3	0.9300	C25—H25A	0.9600
C2—C1	1.371 (3)	C25—H25B	0.9600
C2—H2	0.9300	C25—H25C	0.9600
C1—C6	1.392 (3)	C20—C19	1.388 (4)
C1—H1	0.9300	C20—H20	0.9300
C6—C5	1.383 (3)	C19—H19	0.9300
C6—C7	1.472 (3)	C10—C11	1.486 (3)
C7—C8	1.503 (3)	C11—C12	1.380 (3)
C8—C9	1.333 (3)	C11—C16	1.386 (3)
C9—C10	1.458 (3)	C16—C15	1.372 (4)
C9—C17	1.505 (3)	C16—H16	0.9300
C17—C18	1.516 (3)	C15—C14	1.365 (5)
C17—H17	0.9800	C15—H15	0.9300
C18—C19	1.374 (3)	C14—C13	1.372 (5)
C18—C23	1.379 (3)	C14—H14	0.9300
C23—C22	1.384 (3)	C13—C12	1.372 (4)
C23—H23	0.9300	C13—H13	0.9300
C22—C21	1.362 (4)	C12—H12	0.9300
C22—H22	0.9300	C5—C4	1.385 (3)
C21—C20	1.365 (5)	C5—H5	0.9300
C21—H21	0.9300	C4—H4	0.9300
C24—C25	1.514 (3)		
			
C8—O2—N1	107.60 (14)	C24—C26—H26B	109.5
O2—N1—C17	105.62 (13)	H26A—C26—H26B	109.5
O2—N1—C24	105.59 (14)	C24—C26—H26C	109.5
C17—N1—C24	116.51 (17)	H26A—C26—H26C	109.5
C2—C3—C4	120.6 (2)	H26B—C26—H26C	109.5
C2—C3—H3	119.7	C24—C27—H27A	109.5
C4—C3—H3	119.7	C24—C27—H27B	109.5
C3—C2—C1	120.3 (2)	H27A—C27—H27B	109.5
C3—C2—H2	119.9	C24—C27—H27C	109.5
C1—C2—H2	119.9	H27A—C27—H27C	109.5
C2—C1—C6	120.1 (2)	H27B—C27—H27C	109.5
C2—C1—H1	119.9	C24—C25—H25A	109.5
C6—C1—H1	119.9	C24—C25—H25B	109.5
C5—C6—C1	119.32 (19)	H25A—C25—H25B	109.5
C5—C6—C7	122.32 (18)	C24—C25—H25C	109.5
C1—C6—C7	118.14 (19)	H25A—C25—H25C	109.5
O1—C7—C6	122.5 (2)	H25B—C25—H25C	109.5
O1—C7—C8	117.9 (2)	C21—C20—C19	120.3 (3)
C6—C7—C8	119.47 (18)	C21—C20—H20	119.9
C9—C8—O2	114.52 (17)	C19—C20—H20	119.9
C9—C8—C7	134.23 (18)	C18—C19—C20	120.4 (3)
O2—C8—C7	111.20 (16)	C18—C19—H19	119.8
C8—C9—C10	130.56 (18)	C20—C19—H19	119.8
C8—C9—C17	108.23 (16)	O3—C10—C9	119.5 (2)
C10—C9—C17	121.17 (17)	O3—C10—C11	120.21 (18)
N1—C17—C9	103.90 (15)	C9—C10—C11	120.22 (18)
N1—C17—C18	108.96 (15)	C12—C11—C16	119.6 (2)
C9—C17—C18	113.33 (16)	C12—C11—C10	120.88 (19)
N1—C17—H17	110.2	C16—C11—C10	119.3 (2)
C9—C17—H17	110.2	C15—C16—C11	119.5 (3)
C18—C17—H17	110.2	C15—C16—H16	120.2
C19—C18—C23	118.5 (2)	C11—C16—H16	120.2
C19—C18—C17	121.1 (2)	C14—C15—C16	120.7 (3)
C23—C18—C17	120.37 (19)	C14—C15—H15	119.6
C18—C23—C22	120.9 (2)	C16—C15—H15	119.6
C18—C23—H23	119.6	C15—C14—C13	119.9 (3)
C22—C23—H23	119.6	C15—C14—H14	120.1
C21—C22—C23	120.0 (3)	C13—C14—H14	120.1
C21—C22—H22	120.0	C14—C13—C12	120.2 (3)
C23—C22—H22	120.0	C14—C13—H13	119.9
C22—C21—C20	119.9 (2)	C12—C13—H13	119.9
C22—C21—H21	120.0	C13—C12—C11	120.0 (3)
C20—C21—H21	120.0	C13—C12—H12	120.0
N1—C24—C25	113.87 (17)	C11—C12—H12	120.0
N1—C24—C27	105.9 (2)	C6—C5—C4	119.8 (2)
C25—C24—C27	110.46 (19)	C6—C5—H5	120.1
N1—C24—C26	106.08 (17)	C4—C5—H5	120.1
C25—C24—C26	110.6 (2)	C3—C4—C5	119.9 (2)
C27—C24—C26	109.7 (2)	C3—C4—H4	120.1
C24—C26—H26A	109.5	C5—C4—H4	120.1
			
C8—O2—N1—C17	−3.35 (19)	C17—C18—C23—C22	180.0 (2)
C8—O2—N1—C24	120.65 (17)	C18—C23—C22—C21	0.9 (4)
C4—C3—C2—C1	1.4 (4)	C23—C22—C21—C20	−0.2 (5)
C3—C2—C1—C6	−0.3 (4)	O2—N1—C24—C25	−58.2 (2)
C2—C1—C6—C5	−1.0 (3)	C17—N1—C24—C25	58.7 (2)
C2—C1—C6—C7	173.7 (2)	O2—N1—C24—C27	63.41 (19)
C5—C6—C7—O1	158.6 (2)	C17—N1—C24—C27	−179.74 (18)
C1—C6—C7—O1	−15.9 (3)	O2—N1—C24—C26	179.99 (18)
C5—C6—C7—C8	−17.5 (3)	C17—N1—C24—C26	−63.2 (2)
C1—C6—C7—C8	167.99 (18)	C22—C21—C20—C19	−0.3 (4)
N1—O2—C8—C9	1.9 (2)	C23—C18—C19—C20	0.3 (3)
N1—O2—C8—C7	179.93 (15)	C17—C18—C19—C20	179.5 (2)
O1—C7—C8—C9	121.7 (3)	C21—C20—C19—C18	0.3 (4)
C6—C7—C8—C9	−62.0 (3)	C8—C9—C10—O3	162.4 (2)
O1—C7—C8—O2	−55.8 (3)	C17—C9—C10—O3	−14.9 (3)
C6—C7—C8—O2	120.48 (19)	C8—C9—C10—C11	−19.3 (3)
O2—C8—C9—C10	−177.13 (19)	C17—C9—C10—C11	163.36 (17)
C7—C8—C9—C10	5.4 (4)	O3—C10—C11—C12	124.2 (2)
O2—C8—C9—C17	0.4 (2)	C9—C10—C11—C12	−54.0 (2)
C7—C8—C9—C17	−177.0 (2)	O3—C10—C11—C16	−50.4 (3)
O2—N1—C17—C9	3.45 (18)	C9—C10—C11—C16	131.4 (2)
C24—N1—C17—C9	−113.39 (17)	C12—C11—C16—C15	0.2 (3)
O2—N1—C17—C18	−117.64 (17)	C10—C11—C16—C15	174.8 (2)
C24—N1—C17—C18	125.52 (18)	C11—C16—C15—C14	−2.3 (4)
C8—C9—C17—N1	−2.5 (2)	C16—C15—C14—C13	2.2 (5)
C10—C9—C17—N1	175.33 (17)	C15—C14—C13—C12	0.0 (4)
C8—C9—C17—C18	115.60 (19)	C14—C13—C12—C11	−2.1 (4)
C10—C9—C17—C18	−66.6 (2)	C16—C11—C12—C13	2.0 (3)
N1—C17—C18—C19	−111.1 (2)	C10—C11—C12—C13	−172.5 (2)
C9—C17—C18—C19	133.7 (2)	C1—C6—C5—C4	1.2 (3)
N1—C17—C18—C23	68.0 (2)	C7—C6—C5—C4	−173.24 (19)
C9—C17—C18—C23	−47.1 (3)	C2—C3—C4—C5	−1.2 (4)
C19—C18—C23—C22	−0.9 (4)	C6—C5—C4—C3	−0.1 (3)

### Hydrogen-bond geometry (Å, º)

*Cg*1 is the centroid of the C18–C23 ring.

**Table:**

*D*—H···*A*	*D*—H	H···*A*	*D*···*A*	*D*—H···*A*
C3—H3···O3^i^	0.93	2.51	3.203 (3)	131
C25—H25*A*···O1^ii^	0.96	2.59	3.483 (3)	155
C2—H2···*Cg*1^i^	0.93	2.70	3.490 (3)	143

Symmetry codes: (i) −*x*+1/2, *y*+1/2, *z*−1/2; (ii) *x*, *y*, *z*+1.
